# Advancing transcriptomic profiling of airborne bacteria

**DOI:** 10.1128/aem.00148-25

**Published:** 2025-04-28

**Authors:** Emily Antoinette Kraus, Bharath Prithiviraj, Mark Hernandez

**Affiliations:** 1 Environmental Engineering, University of Colorado Boulder 129263, Boulder, Colorado, USA; 2 Microbiome Science Platforms, Reckitt Health US, Montvale, New Jersey, USA; 3 Royal Society for Public Health, London, United Kingdom; Shanghai Jiao Tong University, Shanghai, China

**Keywords:** bioaerosol, transcriptomics, mRNA, airborne microorganisms, aerobiome

## Abstract

Aerobiology research focusing on bioaerosol particle dynamics has catalogued the identity, distribution, and abundance of airborne microbes in a broad variety of indoor environments and, more recently, indoor disinfection methods for medically relevant microbes. Given their importance in environmental health and our constant exposure to airborne microbes in our daily lives, surprisingly little is known about the activity of live bioaerosols and their metabolic responses to aerosolization and suspension stress. In this context, microbial messenger RNA (mRNA) is a powerful information source of near-real-time organismal responses that cannot be attained through genomic, proteomic, or metabolomic studies. This review discusses current knowledge from transcriptomic studies describing airborne bacterial cellular activity in response to a myriad of environmental stresses imparted rapidly upon aerosolization and continued suspension as a microscopic bioaerosol. In the context of transcriptome profiling, potential artifacts associated with aerosolization/collection of bioaerosols are discussed from the perspective of preserving mRNA and maintaining its fidelity as it exists in airborne microbes. Recommendations for advancing live bioaerosol metabolic profiling through gene expression studies are presented to mitigate inherent artifacts and challenges with modern bioaerosol experiments. These recommendations include the use of larger experimental chambers, temperature control during aerosolization processes, and liquid capture bioaerosol sampling into a nucleic acid preservative to improve the fidelity of collected RNA and better capture the transcriptional activity of airborne microorganisms. Eventually, improvements in profiling bioaerosol activity can contribute toward answering fundamental questions on the aerobiome such as: is the atmosphere a temporary highway or a habitat for microorganisms?

## CURRENT PARADIGMS OF MICROBIAL FUNCTION AND PERSISTENCE IN AIR

Airborne microorganisms are found all around us, indoors and out. Airborne microbes and non-living biological particles (bioaerosols) are central to environmental health and climate processes (1). Atmospheric bioaerosols can spread allergens, toxigenic agents, and infectious disease, act as cloud condensation and ice nuclei (2, 3), and may impact carbon speciation within clouds (4, 5). Within the built environment, bioaerosol inhalation is a primary route of microbial exposures, in addition to those associated with surface fomites and water sources (6). Although treated water and fomites have been extensively studied as routes of microbial exposure for humans, bioaerosols are a relatively understudied route of great potential importance to environmental health (6). Within indoor environments, studies have examined the microbial composition and viability of aerosolized organisms (6), sources and relative fluxes of bioaerosols (7), their genomic functional capabilities, and transcriptional activity interpreted from DNA and messenger RNA (mRNA) sequencing. With the growing body of bioaerosol dynamics research, the metabolic activities employed by airborne organisms to survive within an aerosol are gaining increased attention. Compiling and deciphering mechanistic responses underpinning microbial survival in air are indispensable toward understanding their persistence in an airborne state, indoors or out, and may assist in designing better engineering interventions that can selectively inactivate live bioaerosols in relevant scenarios.

**Fig 1:**
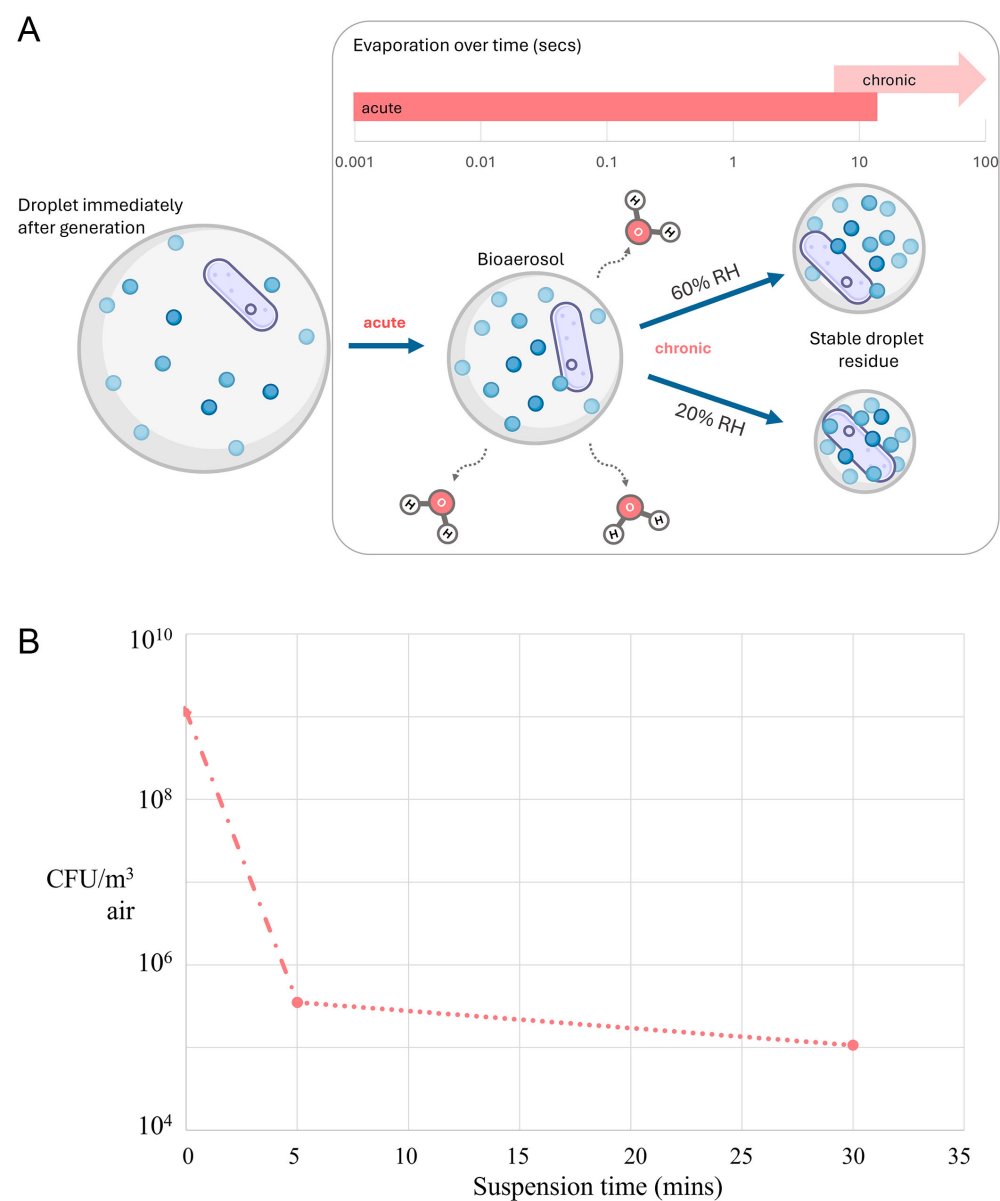
(A) Schematic of bioaerosol droplet evolution to a stable aerosol nucleus under two relative humidity (RH) conditions commonly encountered indoors. Acute aerosolization stress and chronic suspension stress phases are highlighted over time. Evaporation is dependent on RH, temperature, droplet initial size, and droplet composition. Adapted from reference 14 with permission. (B) Example recovery of culturable *Pseudomonas syringae* showing the effects of acute and chronic aerosolization stress. *P. syringae* was aerosolized in a Collison nebulizer at 20 psi and 48 L/min air flow into an unventilated, well-mixed 10 m^3^ chamber at 60% RH. Bioaerosols were collected with a Biospot VIVAS condensation capture sampler at a flow rate of 8 L/min.

Airborne microorganisms are exposed to a myriad of unique environmental stressors during aerosolization and suspension that impact their metabolism and persistence in air. These stressors include humidity (8), temperature, light exposure, oxidative stress (9), osmotic stress within aerosol droplets (10, 11), efflorescence (9, 12), and atmospheric pollutants/particulates (13). There are two distinct phases of stressors as microorganisms become airborne. First, organisms can undergo acute aerosolization stress during their progression from aqueous, sessile, or soil environments into air as they are subject to rapid changes in their localized environment immediately after partitioning into a bioaerosol. This initial aerosolization can include evaporation processes postulated to occur within milliseconds to 10 s of seconds as microbes progress from an aqueous environment to the atmospheric environment (Fig. 1A) (14). In prolonged suspension, organisms experience a distinctly different phase of chronic airborne stress as they remain altered and age in the atmospheric environment, which can continue over much longer time scales than acute aerosolization stress, on the order of minutes, hours, or even days (Fig. 1A and B). While we know through numerous culturing and viability studies that these phases and factors impact the ability of live bioaerosols to persist, there is a paucity of studies examining how bacteria respond to being airborne on a fundamental metabolic level. This review summarizes the current knowledge of airborne activity of live bacterial bioaerosol transcriptomic studies and offers recommendations to improve RNA-based observations in the emerging generation of bioaerosol experiments.

Early aerobiome work envisioned the atmosphere as an inhospitable environment for microbial life wherein airborne microbes were metabolically inactive (e.g., spores) or otherwise lost functionality soon after entering an airborne state due to composite stresses (15–17). Droplet modeling and contemporary experimentation indicate that artificially produced aerosol droplets will evaporate rapidly in air as they become stable droplet nuclei (18–20); the kinetics to stability is relative to master aerosol variables of aerodynamic diameter, moisture content, viscosity, surface activity, pH, and oxidation state. Acknowledging that intact individual cells may themselves qualify as “an aerosol,” for any microorganisms associated with microdroplets, the aerosolization and suspension process can be highly stressful, as temperature, water content, and solute concentrations change rapidly, imparting osmotic and desiccation stress as phase changes or amorphous vitrification occur in the aerosol (11, 21, 22). Despite the immediate environmental challenges microbes experience as bioaerosols, evidence of metabolism and even airborne replication of culturable bacteria was documented in the 1970s (23, 24), and increasing evidence of microbial activity in an airborne state has emerged in recent years. For example, incubations of environmental isolates from cloud water demonstrate bacterial growth and degradation of chemical components common within clouds (carboxylic acids, formaldehyde, and methanol) (25, 26), which is sustained in the presence of common atmospheric oxidants (e.g., hydrogen peroxide) (5) and cold temperatures (5°C) typical of low-altitude clouds (4). Activity of photolyase enzymes that repair DNA lesions caused by UV radiation of airborne *Mycobacterium parafortuitum* was established by chamber suspension experiments, and the level of induced intracellular enzymatic activity correlated to the imparted UV dose and humidity (27). Airborne *Sphingomonas aerolata* was shown to be transcriptionally active and responsive to gaseous substrates based on the RNA:DNA ratio of 16S genes extracted from their cells captured in liquid impingers (28). Therefore, our understanding of the aerobiome has shifted to recognize aerosol droplets as temporary microenvironments containing limited nutrients and water for organisms aloft in the atmosphere and built environment, wherein metabolism is driven largely by the need to acclimatize to rapidly changing environmental conditions (29).

**Fig 2:**
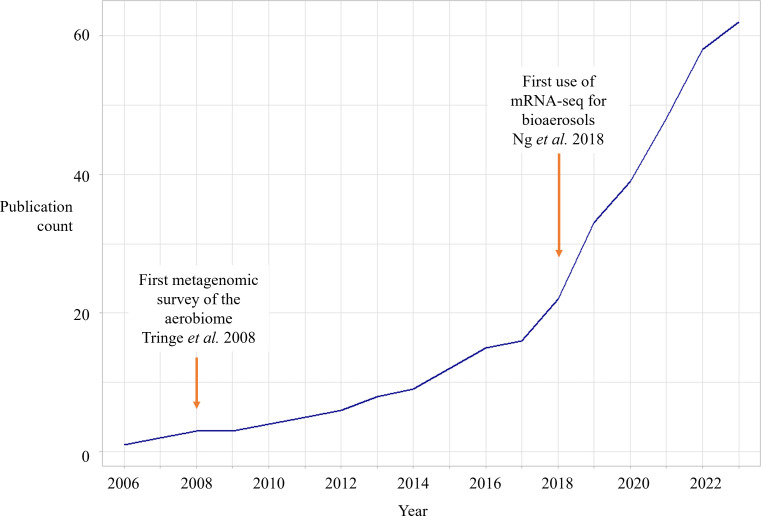
Peer-reviewed publications, including metagenomic characterizations of metabolic functioning of bioaerosols, since 2006.

With the emergence of improved molecular methods for microbiome exploration, publications on bioaerosol metabolic activity in the last two decades have increased substantially, as shown in Fig. 2. DNA- and RNA-based molecular assays have allowed researchers to access a diverse spectrum of the airborne microbiome (often called the “aerobiome”) in ways culture-based methods cannot, given that >99% of bacteria identified by 16S rRNA sequences in atmospheric cloud water samples were not recoverable by conventional culture (26). Metagenomics, amplicon DNA sequencing, or quantification of 16S rRNA genes, antibiotic resistance genes, or other marker genes have been reported in studies to infer information on aerosolized organism metabolic capabilities through their genomes (Table S1). Of the publications investigating bioaerosol metabolic activity in Fig. 2, the majority (57 out of 72) employ these DNA-centric molecular methods to infer activity (Fig. S1). DNA-centric analyses have also illuminated the distribution and diversity of antibiotic resistance genes in a wide variety of environments, notably including indoor and outdoor aerosols (30, 31) (Table S1). Despite the challenges, metagenomic studies of airborne bacteria have recovered and identified aerobiome genes associated with dormancy and sporulation, cold shock resistance, response to oxidative and osmotic stress, antibiotic resistance, repair of UV-induced damage, as well as iron and nitrogen metabolism (32) (Table S1). Such DNA-based studies are limited in their interpretive power, as 16S rRNA transcripts and genes are much more stable over time than mRNA molecules, hindering this approach as a time-resolved indicator of airborne metabolic activity (33). Metagenomic investigations can thus only indiscriminately provide information about the functional gene potential of all live, dormant, and dead cells within a sample, and the aerobiome is no exception.

To ascertain metabolic activity within a sample at the time of collection, high-throughput mRNA sequencing can be used to generate a transcriptome, provided that transcriptome can be preserved, as it was recovered from the environmental source—in this case aerosols, which have notoriously low levels of both bacterial biomass as well as mRNA pools. The mRNA contents of a cell can describe the near-real-time environmental stressors encountered since mRNA is produced as-needed and has a rapid turnover given the average half-life of this transcription intermediate is ≤5 min in classic experiments with *Escherichia coli* and *Bacillus subtilis* (34). Messenger RNA sequencing is now a commonplace tool for characterizing microbial activity in a variety of environments, including those associated with indoor environment exposures, such as treated water and surface fomites (Table S2). However, transcriptome-based gene expression studies of airborne organisms are difficult due to the low cell densities commonly encountered in the atmosphere and many indoor spaces (estimated range for bacteria between 10^4^ and 10^6^ cells/m^3^ air) (35, 36) and the limited ability of aerosol samplers to conserve organisms’ membrane and/or genomic integrity while concentrating airborne microbial biomass (37). If these collection challenges can be overcome, transcriptome analyses can confidently link specific gene expression profiles to environmental stressors in a time-resolved manner, which is particularly useful for airborne organisms as they undergo rapid environmental changes upon aerosolization and again upon their recovery from air (37, 38).

## GENE EXPRESSION CHANGES OF BACTERIA IN RESPONSE TO AIRBORNE SUSPENSION

At the time of this review, at least five studies have examined metabolic responses of bacteria via mRNA gene expression in response to becoming and remaining airborne from an aqueous environment. In these and other bioaerosol studies, “aerosolization” is the rapid, forced partitioning of bacteria from any medium into an atmospheric environment, and “nebulization” is a method of aerosolization of particles from a liquid medium. Ng and coauthors (11) were the first to use transcriptomics, in addition to mutant strains of *E. coli* and aerosol droplet modeling, to study bacterial persistence in air. These authors showed that as an aerosolized droplet containing a bacterium rapidly evaporates toward local equilibrium, these airborne bacteria experience a high degree of environmental change and metabolic stress as judged by transcriptome analyses.

Ng and colleagues reported that counts of differentially expressed genes (DEGs) in the nebulization, aerosolization, and suspension phases were 7, 65, and 1, respectively; indicating the greatest need for the bacteria to respond to change was during an acute aerosolization phase (11). During controlled nebulization, the gene expression profiles of *E. coli* demonstrated an upregulation of multiple genes involved in the cold shock response and the downregulation of the *acs* gene, potentially indicating a reduction of energy metabolism. At the point of initial aerosolization, genes encoding proteins assumed to mitigate hyperosmotic stress response (*osmY*), universal stress response (*uspBG*), protective DNA-binding in nutrient-limited cells (*dps*), cell regulation in stationary phase (*ecnB*, *vspB*, *ygiB*), and outer membrane stabilization during stationary phase (*slp*) were upregulated. Transcripts encoding a sorbitol transporter (*srlA*) gene were found upregulated as well, which the authors attribute as a possible response to nutrient limitation, osmotic stress, and/or cold shock. DEGs downregulated during aerosolization included those for cold shock proteins, colony formation and cell aggregation (*flu*), heat shock proteins (*ibpB*), and pore formation for outer membrane diffusion (*ompF*). After 30 min aloft, only one differentially expressed gene, *ypfM* of unknown function, was downregulated in the airborne *E. coli* collected onto a 0.22 µm filter. This relative activity reduction and downregulation at 30 min in this study may suggest *E. coli* enters a dormant stage, during which protein synthesis is limited to essential proteins for cellular survival. To assess the importance of several DEGs to the survival of *E. coli*, knockout mutant strains were aerosolized in subsequent testing. Strain mutants with nonfunctional *dps* (DNA protection during starvation) or *srlA* genes could not persist during airborne suspension, as well as the corresponding parental strain with a 0.2–0.45 log reduction (11). However, this *srlA* sorbitol transporter gene is also linked to cold stress response, which may be associated with the imparted cold shock experienced during liquid nebulization. Overall, findings within this experimental framework could be further validated in the context of its experimental design. Specifically, the influence of aerosolization on transcription response cannot be unequivocally distinguished from the influence of collision nebulization (evaporative cooling and mechanical stresses) and the subsequent impaction process without paired controls. The observation of 65 DEGs during the aerosolization phase may reflect an increased bacterial response to environmental changes; however, this DEG response could also be attributed to collection stresses from impaction onto a dry filter at relatively high velocity (28 L/min). Complementary strains for these knockouts were not used to assess the stability of the mutants, so the altered phenotype observed in these experiments cannot be isolated with respect to the specific gene mutation; thus, the mutation’s secondary effects on cell physiology during its growth or the atmospheric environment cannot be ruled out. The overwhelming lack of DEGs in *E. coli* during 30 min of suspension warrants further investigation, as this finding may be an experimental artifact due to the use of stationary phase (less metabolically active) bacteria for aerosolization. While some bacterial responses were unresolved, these initial insights into bioaerosol transcriptomics demonstrated that bacteria respond to aerosolization conditions and that mRNA sequencing can be successfully applied to bacteria recovered from air samples, representing a significant step forward in the study of bioaerosol activity. This series of bioaerosol investigations highlights a key point for aerobiology studies where genetically engineered bacteria are concerned: as parallel control, complementary strains of genetic “knockouts” should always accompany any observations of airborne mutant behaviors, such that a purposely altered phenotype can be isolated with respect to any specific gene mutation.

Three studies have examined cloud water and clear sky metatranscriptomes from Puy de Dôme, a high-altitude research station in France. In a targeted 2018 study, Lallement and coauthors identified transcripts of phenol monooxygenase and hydroxylase and catechol 1,2-dioxygenase as potential evidence for microbial phenol degradation within three samples of cloud water. The phenol degradation transcripts were associated with several *Acinetobacter* and *Pseudomonas* strains, and culture experiments with cloud water isolates supported phenol metabolism (39). In 2019, Amato and colleagues published a report of comparative metatranscriptomics of bacteria collected from the same site at night, finding their metabolic responses converged around responding to cold, oxidants, and osmotic changes when compared to bacteria from other environments (29). The transcription profile indicating a response to cold temperatures was found as the expression of specific genes involved in membrane alteration, amino acid metabolism, and redox cofactor synthesis/transport. Transcripts and genes encoding proteins for catalase, superoxide dismutase, peroxiredoxin, peroxidase, and antioxidant compound synthesis indicate a bacterial response to oxidant and free radical detoxification, as well as maintenance of cell homeostasis. Expression of genes for iron and copper transport and siderophore synthesis/transport indicated possible iron scavenging activity in this aerobiome, and transcripts related to extracellular polymeric substance production and cell adhesion indicate a potential for promoting cell aggregation induction. Evidence of metabolism of single-carbon compounds was denoted by transcripts for tetrahydrofolate synthesis, possibly pointing toward methylotrophy in the atmosphere. The microbial community identified in the metatranscriptome sequences included members of Alphaproteobacteria, Betaproteobacteria, Planctomycetes, Chlorobia, and Cyanobacteria, providing some insight into the transcriptionally active community within low-altitude clouds. While this study was not able to differentiate between acute aerosolization stress and chronic suspension stress, it revealed some mechanistic pathways for survival that may be used by atmospheric bacteria on global scales. Most recently, in the third study from Puy de Dôme, Peguilhan et al. compared metatranscriptomes and metagenomes from samples of clouds and clear sky, with the findings of upregulated transcripts in the atmospheric aerobiome as a whole being similar overall to Amato et al. (29, 40). Notably, the authors report the ratio of RNA:DNA in clouds is higher than that of clear sky, suggesting a higher relative level of metabolic activity in cloud conditions. They posit that the increase in water availability within the clouds may allow for an increase in microbial activity, reminiscent of the “Birch effect” in rewetted dry soils.

Recent work by Barnes and Wu (22) aerosolized *Klebsiella pneumoniae* in synthetic saliva into a small 118 L chamber held under four different temperature and humidity combinations to examine airborne survival mechanisms. Though no aerosol sample was taken directly after nebulization, the authors found a total of 201 DEGs in *K. pneumoniae* in the nebulizer after nebulization and 132 DEGs after 15 min of airborne suspension under the four conditions observed. These counts generally agreed with Ng and colleagues’ observation of higher DEGs numbers in *E. coli* during the initial aerosolization. In the source bacterial culture retained after 5 min of nebulization, genes for iron sequestration (*entF*), fatty acid oxidation (*fadAB*), ATP synthesis and electron transport (*nuoLN*), and phenyl acetic acid degradation were upregulated, while genes for peptidoglycan binding (*lysM*), fimbrial proteins, and lipid A synthesis were downregulated. Aerosolized *Klebsiella* DEGs included evidence of altered membrane fluidity and rigidity by upregulation of genes involved in fatty acid degradation, sphingolipid metabolism, and of biosynthesis of glycosphingolids. The authors interpreted this membrane alteration profile to a possible cold adaptation response. Metabolic pathways indicating the cells potentially experienced nutrient-starved conditions, such as the β-oxidation pathway, maltose system, gluconeogenesis, and ethanolamine utilization (*eut*), were upregulated during airborne suspension. Genes for microcompartment formation, antibiotic resistance, and those potentially involved in host infection and/or virulence (*eut*) were upregulated in the *Klebsiella*.

**Fig 3:**
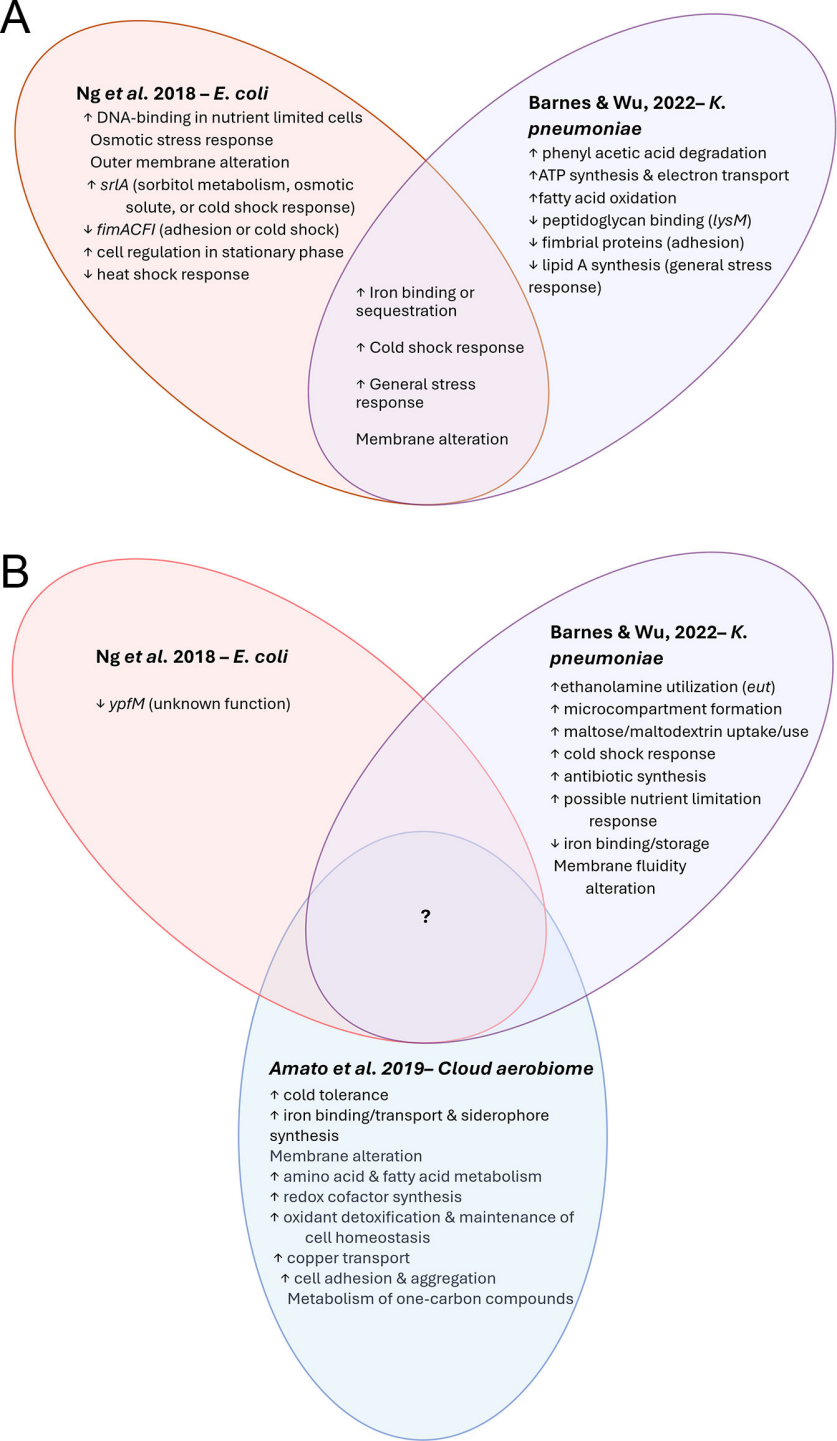
DEGs identified during phases of (A) acute aerosolization stress and (B) chronic suspension stress phases from the untargeted transcriptomics studies on bacterial stress response in air. ↓ = downregulated DEGs, ↑ = upregulated DEGs. No arrow indicated multiple related DEGs were upregulated and downregulated (22). There was no aerosol sample directly after nebulization; thus, the acute phase was solely nebulization-induced stress identified from a liquid sample (39). This was a targeted analysis centered on phenol degradation and, thus, was not included.

These innovative studies begin to provide a better fundamental understanding of bacterial metabolism immediately after entering air from an aqueous environment and subsequently enduring longitudinal air suspension for several minutes. Several trends in the gene expression profiles are apparent from the limited studies observing bioaerosol transcriptomes (Fig. 3). During the acute aerosolization phase of chamber studies on *E. coli* and *K. pneumoniae* (Fig. 3A), transcripts related to iron binding, storage, and siderophore synthesis are noted in three of the experiments, indicating iron uptake and metabolism may be important for bacterial persistence in air. Chamber experiments identify a strong cold shock response, the strength of which, however, may be an artifact of uncontrolled nebulizer cooling during aerosolization. A generalized stress response to desiccation, osmotic stress, and oxidative stress is observed, which changes with temperature and humidity conditions. Gene expression shifts are related to membrane structure and function, suggesting aerosolization induces alterations to bacterial membrane fluidity and rigidity. Gene expression profiles of aerosolized isolates and atmospheric microbes entrained in longer airborne suspension show no definitive overlap in metabolic activity, as shown by the central portion of Fig. 3B. This paucity of information (i) highlights the relative infancy of bioaerosol research and (ii) this fundamental gap in our understanding of survival mechanisms airborne microorganisms employ in prolonged atmospheric suspension and (iii) provides a key target for characterization in future environmental and lab-based bioaerosol investigations.

## TECHNOLOGICAL ADVANCEMENTS TO FACILITATE HIGH-FIDELITY MOLECULAR CHARACTERIZATION OF BIOAEROSOLS

**Fig 4:**
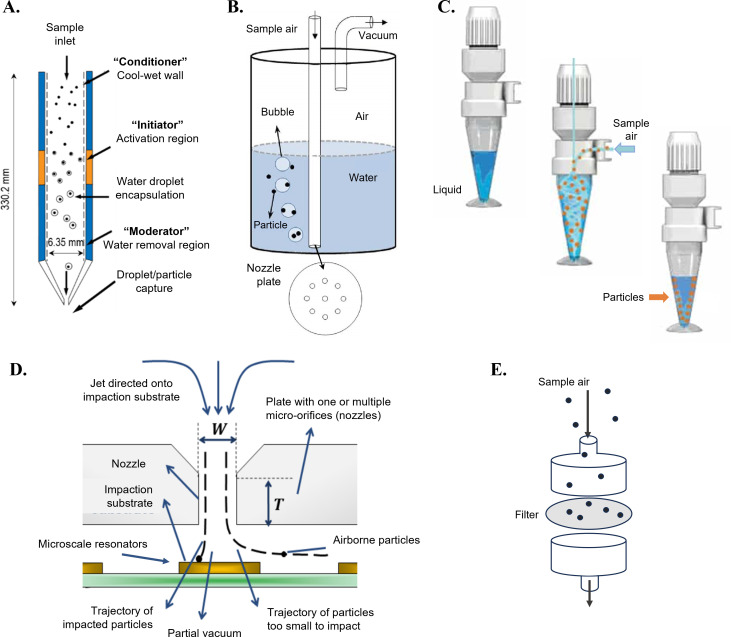
Comparison of sampler types commonly used for collection and characterization of bioaerosols, including (A) condensation capture into liquid (Aerosol Devices, Inc., Fort Collins CO, USA) (reprinted from reference 38 with permission); (B) capture with an impinger (e.g., the SKC biosampler) into liquid (reprinted from reference 44 with permission); (C) capture with a high-air volume wetted wall cyclone collector (Bertin Technologies, France) (image is Bertin Technologies, reprinted from reference 45 with permission); (D) collection onto a solid medium or filter with an impactor (reprinted from reference 46 with permission); and (E) aerosol collection on filters. Liquid capture into a nucleic acid preservative is the best practice to minimize degradation of mRNA from bioaerosols.

For transcriptome profiling, an ideal bioaerosol sampler should (i) collect enough air to ensure that detection thresholds can be met with appropriate time resolution, (ii) concentrate particles for subsequent analyses, and (iii) preserve viability and nucleic acid integrity throughout sampling and storage. Bioaerosol research has primarily relied on conventional sampling devices (shown in Fig. 4) that can compromise cell membrane integrity and/or deliver other significant physiologic collection stresses to live organisms (37, 41). Even brief exposures to common collection stresses can affect bacterial transcription profiles, introducing potential biases due to experimental design factors. Consequently, bioaerosol sampler choice is a critical aspect of the experimental framework and impacts the recovery of genomic material and sequencing results (37, 42, 43).

Cells collected by filtration and impaction onto solid media (filters, gelatin, etc.) experience mechanical and desiccation stress as liters of air pass through a device at relatively high velocities, whereas liquid capture via impingers prevents dehydration but facilitates cold stresses during sampling because of the latent heat losses associated with evaporation. Depending on impinger operational conditions—local humidity, air temperature, and impinger flow rates— impinger temperatures drop markedly below ambient conditions; often these impinger reservoirs approach refrigeration levels (below 10°C in 10 min) or even precipitate freezing. Furthermore, liquid impingers induce relatively large pressure drops as particles partition from air to liquid often through a high-velocity orifice. For transcriptomic studies, experimental artifacts are magnified as mRNA changes rapidly in response to altered environmental conditions and are subject to rapid degradation by RNases. Liquid capture samplers are a better choice for mRNA targets due to decreased shear and desiccation stress compared to impaction. Cell cultivability and membrane integrity are better maintained by liquid capture methods like wet walled cyclones and the SKC Biosampler (SKC, Inc., Eighty Four, PA) (37, 47). Though more costly, condensation capture into a liquid genomic preservative has demonstrated promising results as a relatively gentle bioaerosol collection process (38) that does not impart collection stresses that would otherwise induce transcriptome expression during sampling. Commercially available nucleic acid preservatives used in this context are summarized in Table 1. Low cell densities in air also necessitate bioaerosol concentration, which can be directed into a small liquid volume (~0.25–2 mL) with condensation capture devices, some of which include virtual impactors on their intake (air phase particle concentrators) (48). This ‘soft landing’ directly into a liquid preservative (or any liquid or growth medium of choice) is as close as we can currently get to the bioaerosol physiological state *in situ* with available aerosol collection technology. Given the importance of preserving mRNA fidelity, it is essential to carefully evaluate each sampler type to align with the specific requirements of a study and minimize systemic collection stresses and associated biases.

**TABLE 1:** Available nucleic acid preservation solutions for RNA recovery

	Manufacturer	Claims	Cost
DNA/RNA Shield	Zymo Research, Inc.	Stabilizes sample DNA and RNA at ambient temperature for >30 days Somewhat lytic, inactivates sample	$0.96–$1.36 per mL^*a*^
RNAprotect Bacterial Reagent	Qiagen, Inc.	Stabilizes sample RNA to minimize need for immediate freezing, inactivates RNases, and protects expression profile at collection	$1.79^*b*^
RNALater	Life Technologies	Stabilizes sample RNA to minimize need for immediate freezing and inactivates RNases	$1.11–$1.82 per mL^*c*^
TRIzol/TRI Reagent	Life Technologies/ Molecular Research Center, Inc.	Stabilizes RNA, inactivates RNases, aids cell lysis, DNA and proteins can be co-isolated	$2.15–$2.40 per mL^*c*^

^*a*^ Cost data from Zymo Research.

^*b*^ Qiagen.

^*c*^ Thermo Fisher Scientific as of November 2023.

Traditional plate count methods have been historically used to assess airborne bacterial persistence and disinfection response; however, colony enumeration cannot differentiate dead/inactivated cells from viable but non-cultivable cells (49). Additionally, culture media can impart recovery bias for organisms that can grow on a specific media type chosen, and in the case of mixed microbial communities, it is likely that all members within a sample will be unable to grow on a single medium. Given that cell membrane integrity can be damaged by aerosolization and collection methods (37, 41) and thus impact the cultivability of a sample, other options for viable cell quantification are practical. To better determine the total viable (culturable and non-culturable) fraction of organisms in aerosol experiments, propidium monoazide (PMA)-qPCR is a valuable tool (50, 51). PMA is a dye that can enter compromised cell membranes and intercalate DNA backbones when exposed to blue light, preventing the amplification of bound DNA during subsequent PCR or qPCR. PMA does not enter intact cells, allowing for a quick assessment of membrane integrity as a surrogate measure of viability and/or primary physiological damage. The drawback of PMA dye is the potential for differing reaction efficiencies between organisms due to differences in membrane structure, which necessitates additional testing of the efficacy of PMA with a chosen group of organisms and makes PMA better suited for laboratory chamber test regimes.

## RECOMMENDATIONS FOR FUTURE BIOAEROSOL GENE EXPRESSION STUDIES

To advance bioaerosol gene expression studies, several key recommendations are proposed. First, the use of large-scale aerosol chambers (>1 m^3^) with environmental controls can better replicate real-world conditions in indoor spaces like homes and office buildings. Larger chambers also reduce experimental artifacts due to aerosol deposition by ensuring a higher volume-to-surface area ratio and longer particle suspension times, thereby achieving aerosol equilibrium more effectively. Capturing bioaerosols effectively from larger chambers will require either choosing a higher flow rate air sampler, sampling for a longer period of time, or increasing the density of bioaerosol particles within the chamber.

Temperature control during the nebulization process is critical to minimize artificial cooling (due to the latent heat of evaporation) that may induce a cold shock response in live bacterial bioaerosols. Ng and co-authors observed their nebulizer solution temperature drop from 23 to 18°C within 3 min, causing rapid cooling, before the aerosols were exposed to room temperature (~20°C) upon leaving the nebulizer. This rapid cooling (cold shock), followed by extended suspension in warmer air (humidity shock), could explain the downregulation of cold shock transcripts in *E. coli* during the aerosolization phase (11).

To preserve bioaerosol integrity, researchers should prioritize relatively gentle sampling methods, such as condensation capture samplers, which better preserve cellular physiology, RNA integrity, and cultivability (38). In contrast, methods like impaction onto filters can damage cell membranes, reducing culturable recovery (37) while also extending sample processing/handling time that can lead to mRNA degradation over short collection timescales (~5–10 min) (34). Regardless of the sampling approach, the inclusion of recovery controls using calibrated housekeeping genes is essential for the accuracy of quantitative genomic/transcriptomic bioaerosol studies.

When examining pathogenic strains, relative humidity should be controlled within the 40–60% range, as this aligns with typical conditions of the built environment. Additionally, researchers should report the growth stage at which bacterial cells are used in bioaerosol experiments and carefully consider stationary versus exponential growth stage of cells for disinfectant challenge studies. Comparative studies across cellular growth phases can provide valuable insights, as cells likely enter the air at varied growth stages in natural settings.

Short aerosol sampling times (≤5–10 min) are encouraged to capture highly resolved timepoints, given the rapid degradation rates of bacterial mRNA, which can have half-lives of 1–8 min (34). Longer sampling times risk losing temporal resolution, as mRNA signals can respond within 10 s of seconds to sudden changes in conditions. Additionally, avoiding amplification of nucleic acids prior to mRNA sequencing is important to reduce biases when feasible. When low cell densities of air impose biomass limitations for molecular analyses, increasing sample collection is preferable.

Lastly, in experiments involving knockout mutants, complement strains of the mutants should be tested to ensure the genetic modification targeted is directly related to the altered phenotype and does not introduce secondary metabolic disruptions as experimental artifacts. Reductions of overall fitness/survival of a knockout strain in aerosolization experiments must be linked to the target gene studied, as some mutations can lower the overall functionality of a cell in multiple ways.

Functional profiling of microorganisms that leverages gene expression patterns is a powerful tool for understanding microbial responses to environmental challenges, but isolating such patterns as they exist in bioaerosol is a significant challenge when compared to other media. mRNA analyses can help us better understand the atmospheric environment from the perspective of an airborne microbe as they age in aerosols. That perspective, if compiled in a robust catalogue of many different bacterial types, could help elucidate if the atmosphere is a “highway” or “habitat” for various microorganisms (52). Together, these recommendations provide a robust framework for improving reproducibility and biological relevance of bioaerosol gene expression research.

## CONCLUSIONS

The capacity of bacteria to perceive environmental stimuli and generate a rapid mRNA response to physiological stressors offers a powerful analytical tool for aerobiological characterizations if we can adapt aerosol generation and recovery equipment for this purpose without introducing artifacts or significant systematic biases. This review focused on airborne bacteria, as fungal spores and viruses exhibit comparatively diminished reactivity to airborne stimuli. Given that spores and viruses cannot respond in a similar time-resolved manner, bacteria are the *de facto* voice of the microbial aerobiome from a transcriptomic perspective. This disparity raises the question: can insights derived from bacterial mRNA sequencing inform us on the airborne persistence of viruses and fungi? Although it seems apparent that the environmental stressors encountered in the atmosphere will be the same for all microorganisms, their responses to these stressors diverge into passive and active paths. Prospective research may be able to leverage bacterial mRNA responses to elucidate bioaerosol persistence on a broader environmental scale.

Quantitation of microbial responses to environmental stimuli in the atmosphere and indoor air is possible, though difficult, with conventional sampler and sequencing technologies. The conditions associated with aerosolization and aerosol collection can respectively manifest in acute and chronic stress to live bioaerosols, and these artifacts need to be accounted for in the interpretation of results and minimized during experimentation when possible. Practical applications of understanding microorganisms’ transcriptional responses when partitioning into the atmospheric environment are broad, as may be their transcriptional responses to their collection from air. One example is the potential improvement of disinfection/sterilization techniques for pathogens to prevent disease transmission, which is increasingly becoming necessary for antibiotic-resistant organisms, wherein engineering controls on bioaerosols in the built environment can be effective. A better understanding of bioaerosols that are actively metabolizing substrates in the atmosphere can inform on how microbes are potentially impacting global biogeochemical cycles and how microorganisms move across continents and oceans. With our current knowledge and technological advances, we can better catalog and quantify the biological responses, persistence, and survival of “naked” microorganisms and those within aerosol droplets in the greater atmosphere and indoor spaces to garner a better understanding of microbial life in air.

## SUPPLEMENTAL MATERIAL

Supplemental tables aem.00148-25-s0001.xlsx Tables S1 and S2.

Figure S1 aem.00148-25-s0002.docx Publication counts of studies employing various molecular methods to investigate bioaerosol metabolic activity and studies using transcriptomics/metatranscriptomics in three primary exposure routes in the built environment.

ASM does not own the copyrights to Supplemental Material that may be linked to, or accessed through, an article. The authors have granted ASM a non-exclusive, world-wide license to publish the Supplemental Material files. Please contact the corresponding author directly for reuse.
