# Supplementary material for: Advancing transcriptomic profiling of airborne bacteria
Source: Appl Environ Microbiol. 2025 Apr 28;91(5):e00148-25. doi: 10.1128/aem.00148-25 (PMC12093975; doi:10.1128/aem.00148-25)

**Supplemental Information for Advancing transcriptomic profiling of airborne bacteria.**

Emily Antoinette Kraus, Bharath Prithiviraj, Mark Hernandez.

**SI Figure 1**. Publication count of a.) studies employing various molecular methods to investigate bioaerosol metabolic activity. b.) Counts of studies using transcriptomics/metatranscriptomics in three primary exposure routes in the built environment. Citations for these figures are in Tables S1 and S2.


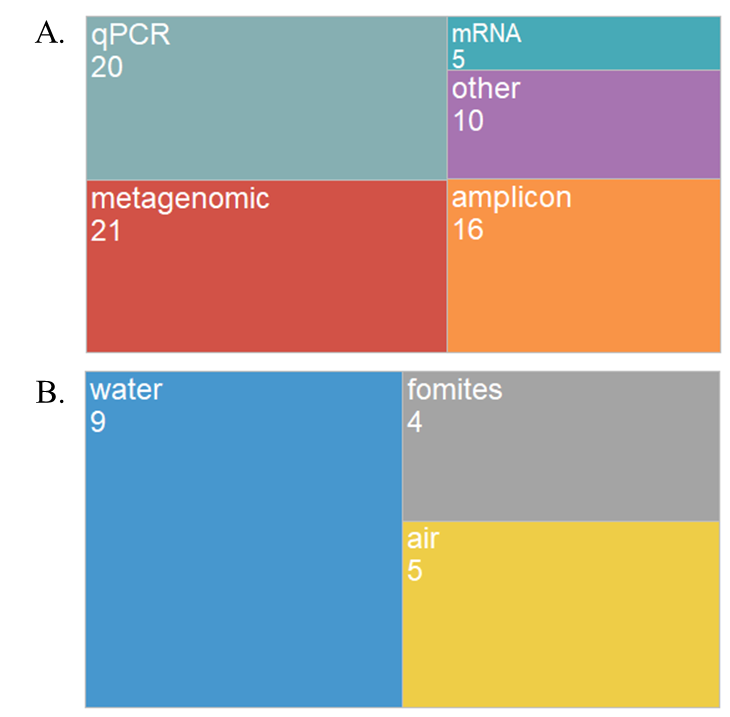

Supplement: Figure S1 — Publication counts of studies employing various molecular methods to investigate bioaerosol metabolic activity and studies using transcriptomics/metatranscriptomics in three primary exposure routes in the built environment. [file aem.00148-25-s0002.docx]
